# Antipsychotic **E**ffects on **C**ortical Morphology in **S**chizophrenia and **B**ipolar **D**isorders

**DOI:** 10.3389/fnins.2020.579139

**Published:** 2020-12-10

**Authors:** Ruiqi Feng, Fay Y. Womer, E. Kale Edmiston, Yifan Chen, Yinshan Wang, Miao Chang, Zhiyang Yin, Yange Wei, Jia Duan, Sihua Ren, Chao Li, Zhuang Liu, Xiaowei Jiang, Shengnan Wei, Songbai Li, Xizhe Zhang, Xi-Nian Zuo, Yanqing Tang, Fei Wang

**Affiliations:** ^1^Department of Radiology, The First Affiliated Hospital of China Medical University, Shenyang, China; ^2^Brain Function Research Section, The First Affiliated Hospital of China Medical University, Shenyang, China; ^3^Department of Psychiatry, Washington University School of Medicine, St. Louis, MO, United States; ^4^Department of Psychiatry, School of Medicine, University of Pittsburgh, Pittsburgh, PA, United States; ^5^Department of Psychiatry, The First Affiliated Hospital of China Medical University, Shenyang, China; ^6^CAS Key Laboratory of Behavioral Science and Research Center for Lifespan Development of Mind and Brain (CLIMB), Institute of Psychology, Beijing, China; ^7^School of Public Health, China Medical University, Shenyang, China; ^8^School of Biomedical Engineering and Informatics, Nanjing Medical University, Nanjing, China; ^9^Key Laboratory of Brain and Education Sciences, School of Education Sciences, Nanning Normal University, Nanning, China

**Keywords:** schizophrenia, bipolar disorder, atypical antipsychotics, magnetic resonance imaging (MRI), cortical thickness

## Abstract

**Background:** Previous studies of atypical antipsychotic effects on cortical structures in schizophrenia (SZ) and bipolar disorder (BD) have findings that vary between the short and long term. In particular, there has not been a study exploring the effects of atypical antipsychotics on age-related cortical structural changes in SZ and BD. This study aimed to determine whether mid- to long-term atypical antipsychotic treatment (mean duration = 20 months) is associated with cortical structural changes and whether age-related cortical structural changes are affected by atypical antipsychotics.

**Methods:** Structural magnetic resonance imaging images were obtained from 445 participants consisting of 88 medicated patients (67 with SZ, 21 with BD), 84 unmedicated patients (50 with SZ, 34 with BD), and 273 healthy controls (HC). Surface-based analyses were employed to detect differences in thickness and area among the three groups. We examined the age-related effects of atypical antipsychotics after excluding the potential effects of illness duration.

**Results:** Significant differences in cortical thickness were observed in the frontal, temporal, parietal, and insular areas and the isthmus of the cingulate gyrus. The medicated group showed greater cortical thinning in these regions than the unmediated group and HC; furthermore, there were age-related differences in the effects of atypical antipsychotics, and these effects did not relate to illness duration. Moreover, cortical thinning was significantly correlated with lower symptom scores and Wisconsin Card Sorting Test (WCST) deficits in patients. After false discovery rate correction, cortical thinning in the right middle temporal gyrus in patients was significantly positively correlated with lower HAMD scores. The unmedicated group showed only greater frontotemporal thickness than the HC group.

**Conclusion:** Mid- to long-term atypical antipsychotic use may adversely affect cortical thickness over the course of treatment and ageing and may also result in worsening cognitive function.

## Introduction

Schizophrenia (SZ) and bipolar disorder (BD) have substantial overlap in genetic vulnerability (The International Schizophrenia Consortium, Purcell et al., 2009; Network and Pathway Analysis Subgroup of Psychiatric Genomics Consortium, 2015), brain structural abnormalities (Goodkind et al., 2015), symptomatology, and cognitive deficits (Tamminga et al., 2013), suggesting a common neurobiological mechanism underlying the two psychiatric conditions. Atypical antipsychotics are first-line treatment options for SZ and BD (Meltzer, 2013) and are effective for treating psychotic symptoms in both disorders (Leucht et al., 2009). They are the mainstay long-term treatment for SZ. Atypical antipsychotics combined with mood stabilizers constitute the mainstay of acute management of bipolar mania and depression and the long-term management of BD (Grande et al., 2016). However, the effects of atypical antipsychotics on cortical structures, especially age-related effects, are still unclear (Goff et al., 2017). Potential effects could have important implications for the course and prognosis of illness.

Previous magnetic resonance imaging (MRI) studies of antipsychotic effects have mainly focused on morphometric changes in cortical volume, thickness and surface area. There may be differences in the short- and longer-term (mid-to-long-term) effects of atypical antipsychotics treatment on cortical structures, such as clinical trials examining the cortical structural changes during short-term (less than 3 months) antipsychotic treatment have reported that cortical thickness is maintained or even increased after 6–12 weeks of antipsychotic treatment (Goghari et al., 2013; Jessen et al., 2018; Nelson et al., 2020), while longer-term (more than 3 months) studies have shown cortical structural loss compared to healthy controls (HC) (Lieberman et al., 2005; Ahmed et al., 2015; Guo et al., 2019; Voineskos et al., 2020). In the largest randomized longitudinal study to date comparing the effects of antipsychotic medication types on brain volumes, olanzapine was found to reduce brain volumes over a 1-year period (Lieberman et al., 2005). In the most recent double-blind, randomized, placebo-controlled trial, major depressive disorder (MDD) patients who were exposed to olanzapine had a significant decrease in cortical thickness across a 6-month period compared with those who took a placebo (Voineskos et al., 2020). Due to the challenges of conducting clinical trials to study atypical antipsychotic effects for more than 1 year, observations mostly come from cross-sectional studies and longitudinal studies with naturalistic designs. For example, cross-sectional studies of SZ patients undergoing current or chronic (over 5 years) treatment suggest cortical loss (Haijma et al., 2013; Lesh et al., 2015; Gjerde et al., 2018; van Erp et al., 2018; Di Sero et al., 2019; Liu et al., 2020). Similar results were observed in BD patients (Gildengers et al., 2014; Birner et al., 2020), although other studies failed to replicate the finding, possibly due to differences in the duration (short, medium and long-term) of atypical antipsychotic treatment and variability in methodology and sample size (Hallahan et al., 2011; Hafeman et al., 2012; Hibar et al., 2018). Longitudinal studies with 3, 7.2, and 9 years of follow-up showed that atypical antipsychotics were associated with progressive cortical structural loss (Ho et al., 2011; Veijola et al., 2014; Akudjedu et al., 2020). However, the findings were not replicated by a meta-regression of longitudinal studies and a 5-year follow-up study (van Haren et al., 2011; Vita et al., 2015). In these studies, a higher mean daily intake or cumulative intake of atypical antipsychotics was associated with less cortical structural loss. Herein, we conduct a preliminary exploratory investigation of the effects of mid- to long-term use of atypical antipsychotics on cortical structures.

Few studies have focused on the effects of atypical antipsychotic on age-related cortical structural changes. Previous studies found greater age-related loss of cortical structures in SZ and BD patients than in HC but were unable to comprehensively examine the influence of antipsychotics (Cropley et al., 2017; Abe et al., 2020). Most studies indicate the absence of antipsychotic effects in the age-related trajectory of cortical measures (Cropley et al., 2017; Altamura et al., 2018), while some show the presence of antipsychotic effects (van Haren et al., 2011). However, evidence regarding the effects of atypical antipsychotics on age-related cortical structural changes in SZ and BD remains uncertain.

Cortical volume is determined by both cortical thickness and surface area, which have distinct genetic influences (Panizzon et al., 2009) and different development trajectories (Wierenga et al., 2014). Thus, we used SBM vertexwise analysis based on cortical surface reconstruction to evaluate thickness and surface area. We opted to combine SZ and BD patients for the following reasons: The criteria for the current prevalent classifications of SZ and BD were established mainly based on clinical symptoms. However, SZ and BD share substantial core features, as indicated by converging lines of evidence from genetic, molecular, histological, and neuroimaging studies (Garcia-Rizo et al., 2016; Goldsmith et al., 2016; Moser et al., 2018; Akudjedu et al., 2020; Writing Committee for the Attention-Deficit/Hyperactivity Disorder et al., 2020). Thus, there appears to be a greater continuum between SZ and BD than previously thought. Previous studies have also selected samples of SZ and BD patients to explore antipsychotic effects (Cross-Disorder Group of the Psychiatric Genomics Consortium, 2013; Ansell et al., 2015; Goodkind et al., 2015). Thus, SZ and BD patients were combined here. To our knowledge, only two studies have investigated how atypical antipsychotics are associated with cortical thickness in SZ and BD. One cross-sectional study did not include an unmedicated comparison group or clear determination of the duration of medication use; it found that SZ and BD patients had thinner medial frontal, parietal and fusiform areas and thicker precentral and postcentral gyri compared to HC (Ansell et al., 2015). One recent naturalistic longitudinal study of psychosis patients with a 3-year follow-up period reported a significantly increased rate of cortical thinning in the left lateral orbitofrontal region compared with HC (Akudjedu et al., 2020). However, evidence of the mid- to long-term effects of atypical antipsychotics is still needed.

Herein, we aimed to determine whether mid- to long-term atypical antipsychotic treatment is associated with cortical structural changes in a real-world observation and, if so, whether age-related cortical structural changes are affected by atypical antipsychotics. We compared cortical thickness and area in SZ and BD patients who had been treated for more than 3 months with atypical antipsychotics, patients who received no psychotropic medication, and HC. We also examined the effects of atypical antipsychotics on clinical symptoms and cognitive function. We hypothesized that regional cortical thickness was more vulnerable to the effects of atypical antipsychotics than surface area was. We also hypothesized that greater cortical thinning would be found in medicated patients than in unmedicated patients and HC and that atypical antipsychotics would affect age-related changes.

## Materials and Methods

### Participants

The study included a total of 445 individuals aged 13–45 years: 88 medicated patients (67 with SZ, 21 with BD) who were treated with atypical antipsychotics for at least 3 months, 84 unmedicated patients (50 with SZ, 34 with BD) who had received no pharmacological treatment for at least 2 months or were medication naive, and 273 HC. Patients were recruited from the inpatient services of the Shenyang Mental Health Centre and the outpatient services of the First Affiliated Hospital of China Medical University. HC participants were recruited from the local community. All participants provided written informed consent after receiving a detailed description of the study. The study was approved by the Institutional Review Board of China Medical University.

All participants were independently assessed by two expert-trained psychiatrists using the Structured Clinical Interview for the Diagnostic and Statistical Manual of Mental Disorders, Fourth Edition (DSM-IV) Axis I Disorders (age ≥18 years) or the Schedule for Affective Disorders and Schizophrenia for School-Age Children-Present and Lifetime version (K-SADS-PL) (age <18 years). All patients met the DSM-IV diagnostic criteria for SZ and BD and had no other comorbid Axis I disorder. HC participants did not have current or lifetime Axis I disorders or a history of psychotic, mood, or other Axis I disorders in first-degree relatives, as determined by detailed family history. HC were matched by age and gender with the medicated and unmedicated patients. Participants were excluded if any of the following were present: (1) substance/alcohol abuse or dependence, (2) concomitant major medical disorder, (3) history of electroconvulsive therapy or transcranial magnetic stimulation therapy, (4) history of head trauma with loss of consciousness for ≥5 min or any neurological disorder and (5) any contraindications for magnetic resonance imaging (MRI).

Symptom severity was assessed by the Hamilton Depression Scale (HAMD), the Hamilton Anxiety Scale (HAMA), the Young Mania Rating Scale (YMRS), and the Brief Psychiatric Rating Scale (BPRS); cognitive function was evaluated by the Wisconsin Card Sorting Test (WCST). Demographic and clinical information are detailed in Table 1.

**TABLE 1:** Demographic, clinical characteristics and cognitive function of healthy controls, the medicated patients, and the unmedicated patients.

Variable	HC (*n* = 273)	Patients (*n* = 172)		*F/*χ*^2^/t*-values	*p*-values
		Medicated patients (*n* = 88)	Unmedicated patients (*n* = 84)		
**Demographic characteristic**					
Age (years)	26.59 (7.15)	25.47 (8.12)	25.11 (8.19)	1.609^$^	0.201
Male	107 (39%)	38 (43%)	34 (40%)	0.443^$^	0.801
Right handedness	253 (94%)	232 (90%)	75 (89%)	3.264^$^	0.515
**Clinical characteristic**					
Illness duration (months)	–	59.20 (59.65)	17.01 (28.52)	30.059^%^	<0.001
First episode, yes	–	39 (44%)	65 (77%)	18.851^%^	<0.001
HAMD Total	(*n* = 251)	(*n* = 74)	(*n* = 65)		
	1.05 (1.71)	5.47 (6.05)	13.40 (9.43)	−5.827^%^	<0.001
HAMA total	(*n* = 250)	(*n* = 69)	(*n* = 60)		
	0.92 (2.11)	3.88 (4.02)	10.93 (9.00)	−5.602^%^	<0.001
YMRS total	(*n* = 245)	(*n* = 63)	(*n* = 60)		
	0.22 (0.83)	2.84 (6.81)	4.30 (8.84)	−1.028^%^	0.306
BPRS total	(*n* = 189)	(*n* = 78)	(*n* = 68)		
	18.41 (1.18)	28.33 (10.41)	34.51 (12.44)	−3.267^%^	0.001
**Cognitive function**					
WCST	(*n* = 194)	(*n* = 53)	(*n* = 47)		
Corrected responses	31.53 (11.54)	20.06 (11.36)	23.87 (12.64)	−1.590^%^	0.115
Categories completed	4.19 (2.09)	2.25 (2.24)	2.66 (2.09)	−0.954^%^	0.342
Total errors	16.55 (11.69)	27.94 (11.36)	24.13 (12.64)	1.590^%^	0.115
Perseverative errors	5.98 (6.98)	10.83 (8.37)	9.68 (11.37)	0.580^%^	0.563
Non-perseverative errors	10.52 (6.54)	17.11 (7.70)	14.49 (8.34)	1.635^%^	0.105
**Medication**					
*Antipsychotic use*					
Duration (months)	–	(*n* = 83) 20.47 (25.55)	–		
Dose OPZ (mg)	–	(*n* = 64) 8.27 ± 6.49	–		
**Antipsychotic type**					
Amisulpride	–	3 (3%)	–		
Aripiprazole	–	24 (27%)	–		
Clozapine	–	12 (14%)	–		
Olanzapine	–	17 (19%)	–		
Paliperidone	–	2 (2%)	–		
Quetiapine	–	17 (19%)	–		
Risperidone	–	33 (38%)	–		
Ziprasidone	–	3 (3%)	–		
Antidepressants (%)	–	15 (17%)	–		
Anticonvulsants (%)	–	17 (19%)	–		

*Data were presented as either n (%) or mean (SD). HC, Healthy Controls; HAMD, Hamilton Depression Scale; HAMA, Hamilton Anxiety Scale; YMRS, Young Manic Rating Scale; BPRS, Brief Psychiatric Rating Scale; WCST, Wisconsin Card Sorting Test. OPZ, Olanzapine equivalent value. ^$^The examination among the medicated, unmedicated, and HC groups. ^%^The examination between the medicated and unmedicated groups.*

### Pharmacological Treatment

We reviewed information on the type, dosage, and duration of medication recorded at the time of the MRI scan. The treatments received reflected real-world clinical practice. According to the existing literature on cortical structure changes following the administration of antipsychotics (Lesh et al., 2015), a treatment duration of more than 3 months would be sufficient to observe brain changes associated with atypical antipsychotic use in our samples. On the basis of existing literature on antipsychotic washout (Garver et al., 2005), we considered subjects who were medication naive or had not taken any psychotropic medication in the 2 months prior to the MRI scanning unmedicated. The mean duration of atypical antipsychotic treatment was 20 months; in 78% (69/88) of participants, the duration was more than 6 months, and 61% (54/88) of the participants, the duration was more than 1 year. In the medicated group, 65 patients took a single atypical antipsychotic, and the other 23 patients took 2 atypical antipsychotics simultaneously. No patients were taking typical antipsychotics, but 15 patients were taking antidepressants (escitalopram, fluoxetine, fluvoxamine, and sertraline) at the time of scanning, and 17 patients were taking anticonvulsants (magnesium valproate and sodium valproate). Doses of antipsychotics were converted to olanzapine equivalents (Leucht et al., 2016). In the unmedicated group, 68 patients were medication naive, and the remaining 16 had discontinued psychotropic medication more than 2 months before the study.

### MRI Acquisition

MRI scans were acquired on a GE signa HDX 3.0T scanner at the First Affiliated Hospital of China Medical University with a standard 8-channel head coil. A 3D fast-spoiled gradient-echo sequence [3D-FSPGR: TR = 7.1 ms, TE = 3.2 ms, matrix = 240 × 240, field of view (FOV) = 24 cm × 24 cm, voxel size = 1 mm ^∗^ 1 mm ^∗^ 1 mm, slice thickness = 1.0 mm without a gap, 176 slices in total, and scan time = 8 min 6 s] was used to obtain sagittal T1-weighted structural images of the whole brain.

### Data Processing

Structural MRI images were processed by the Connectome Computation System (CCS^1^) (Zuo et al., 2013), an integrated informatic platform for multimodal neuroimaging data mining and discovery sciences. Methodological details of the processing have been provided in previous studies (Jiang et al., 2015). Briefly, the CCS processing pipeline employed in the present work included two major parts: (1) volBrain (Manjon and Coupe, 2016) performs noise removal, intensity variationcorrection, and extraction of brain tissues; (2) FreeSurfer (version 6.0^2^) performs cortical surface reconstruction with a series of functions, including brain tissue segmentation, mesh tessellation and deformation to tissue boundaries, surface topological defect correction, and surface inflation into a sphere. All outcomes of the above preprocessing were visually inspected by two researchers, and no participants needed manual editing. Cortical thickness and surface area were calculated in native space. Specifically, the thickness of each vertex was the mean value of twice the shortest calculated distance between the white surface (white–gray interface) and the pial surface (grey-CSF interface) and vice versa. Cortical thickness has been demonstrated to show high test–retest reliability (Madan and Kensinger, 2017). The cortical surface area was derived as the total area of the triangles connected to a vertex. All individual maps of cortical thickness and surface area were smoothed of 10 mm full width at half-maximum (FWHM) using a Gaussian filter and transferred to the standard spherical surface (*fsaverage*).

### Statistical Analysis

Statistical analyses of demographic and clinical characteristics were performed using analysis of variance (ANOVA), two-sample *t*-tests, or χ^2^ tests.

A general linear model (GLM) was applied to examine the vertexwise differences in morphometry, including cortical thickness and surface area, among the three groups for the left and right hemispheres. Age and gender were modeled as covariates of interest. We then tested the effects of the group differences in the two morphometric measurements with statistical correction for multiple comparisons based on the Monte Carlo clusterwise simulation approach, with a p-threshold of 0.001 at the vertex level and a *p*-threshold of 0.01 at the cluster level (Hagler et al., 2006). We also performed vertexwise *post hoc t*-tests for cortical thickness between each pair of groups (medicated vs. HC, unmedicated vs. HC, medicated vs. unmedicated). A three-group comparison of cortical thickness that included intracranial volume (ICV), age and gender as covariates were also performed (see Supplementary Material). Vertexwise analyses of cortical thickness between the diagnostic subgroups were also performed to test for effects of diagnosis: BD/SZ (see Supplementary Material).

For each region showing group-level differences, the mean values of the cortical measures were extracted. *Post hoc* two-sample *t*-tests were applied to test the differences in effects between each pair of groups in these regions. Multiple comparisons were corrected using the false discovery rate (FDR) method, and the significance level was set at *p* < 0.05. The duration of illness was included as a covariate of no interest in the patient subgroup comparison. Multiple regression analyses were used to evaluate the relationships between cortical measures in regions showing group-level differences with 5 potential moderators [diagnosis: BD/SZ, age, gender, atypical antipsychotic use (yes/no), and illness duration] in patients. The 5 moderators were entered concurrently as independent effects, allowing us to examine the magnitude of influence among them, especially illness duration and antipsychotic use. The results were corrected using the FDR method, with *p* < 0.05.

To determine potential relationships between the thickness or surface area of regions showing significant group differences and antipsychotic dosage and duration, symptom severities (HAMD, HAMA, YMRS, and BPRS scores) and cognitive function (WCST scores) in all patients (unmedicated and medicated), exploratory partial correlation analysis controlling for age and gender was performed. The results were corrected using the FDR method, with *p* < 0.05.

To determine the age-related effects on cortical measurements in the three groups, we used the mean values of the cortical measurements in regions with group-level differences and ran a GLM. The cortical measurements were considered dependent variables. Fixed factors included age and group (HC, unmedicated, medicated). Significant interaction effects (age × group) were first examined among the three groups to explore the regions showing age-related alterations and were further disentangled using *post hoc* analysis between each pair of groups to test for differential rates of age-related changes across groups. Additionally, to determine the effects of illness duration on our findings, we investigated the (illness duration × group) interactions. The results were corrected using the FDR method, with *p* < 0.05.

## Results

### Demographic and Clinical Data

Demographic and clinical details are presented in Table 1. We found no significant differences in age, gender or handedness among the three groups. We did observe that the medicated group had significantly lower mean HAMD, HAMA and BPRS scores; longer illness duration; and lower first-episode rates than the unmedicated group (*p* < 0.05). Demographic and clinical details of the SZ and BD patients are shown in Supplementary Table 1.

### Group Differences in Cortical Morphology

**FIGURE 1:**
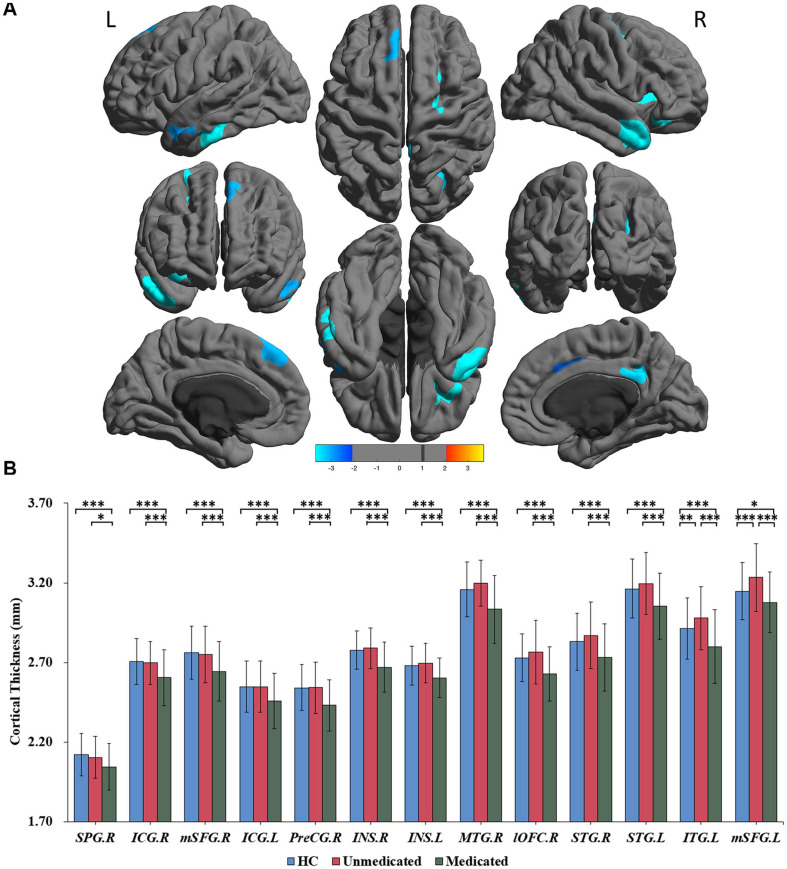
Significant differences in cortical thickness among healthy controls, medicated patients, and unmedicated patients. **(A)** Regions with significant thickness differences among the three groups. The significance level was set at *p* < 0.001 at the vertex level with Monte Carlo clusterwise simulation correction for multiple comparisons (*p* < 0.01, corrected). The color bar represents the *t*-value. **(B)** *Post hoc* pairwise comparisons showing thickness differences between each pairing (HC vs. medicated, HC vs. unmedicated, medicated vs. unmedicated). The significance level was set at *p* < 0.05, with FDR correction for multiple comparisons. ^∗∗∗^*p* < 0.001, ^∗∗^*p* < 0.01, ^∗^*p* < 0.05. HC, healthy controls; R, right; L, left; SPG; superior parietal gyrus; ICG, isthmus of the cingulate cyrus; mSFG, medial superior frontal gyrus; PreCG, precentral gyrus; INS, insula; MTG, middle temporal gyrus; lOFC, lateral orbitofrontal cortex; STG, superior temporal gyrus; ITG, inferior temporal gyrus.

**FIGURE 2:**
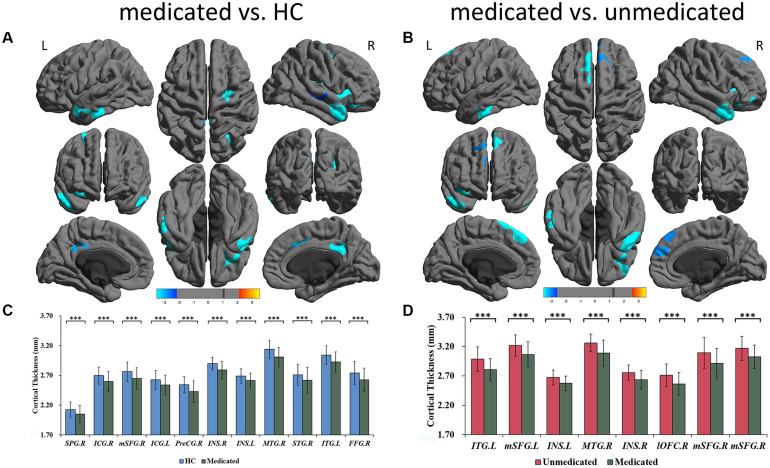
Significant differences of cortical thickness between medicated patients and HC, and between medicated patients and unmedicated patients. **(A)** Regions with significant thickness differences between medicated patients and HC. **(B)** Regions with significant thickness differences between medicated patients and unmedicated patients. The significance level was set to vertex *p* < 0.001 with Monte Carlo cluster-wise simulation correction for cluster *p* < 0.01. L, left. R, right. The color bar represents *t*-value. **(C)** *Post hoc* pair-wise comparisons showing thickness differences between medicated patients and HC. **(D)** *Post hoc* pair-wise comparisons showing thickness differences between medicated patients and unmedicated patients. The significance level was set at *p* < 0.05 with FDR correction for multiple comparison. ^∗∗∗^*p* < 0.001. HC, healthy controls; R, right; L, left; SPG; superior parietal gyrus; ICG, isthmus cingulate gyrus; mSFG, medial superior frontal gyrus; PreCG, precentral gyrus; ISN, insula; MTG, middle temporal gyrus; STG, superior temporal gyrus; ITG, inferior temporal gyrus; lOFC, lateral orbitofrontal cortex; FFG, fusiform gyrus.

A three-group analysis of cortical thickness showed 13 regions with significant group differences (Table 2 and Figure 1A). *Post hoc* analysis showed that compared to the unmedicated and HC groups, the medicated group demonstrated widespread cortical thinning in the bilateral insula, the bilateral isthmus of the cingulate cortex, the bilateral superior frontal gyrus, and the bilateral superior temporal gyrus as well as in the right precentral gyrus, left inferior temporal gyrus, right middle temporal gyrus, right lateral orbitofrontal cortex and right superior parietal gyrus compared with the unmedicated group and HC. Compared to the HC group, the unmedicated patient group showed increased cortical thickness in the left inferior temporal gyrus and the left superior frontal gyrus (Figure 1B). When illness duration was included as a covariate in comparisons of patients (medicated vs. unmedicated), all significant regions except the right parietal cortex survived. We did not detect any significant differences in cortical surface area among the three groups. The results of vertexwise *post hoc t*-tests of cortical thickness were presented in Figure 2 and Supplementary Tables 2, 3. Compared to the HC and unmedicated group, the medicated group showed cortical thinning in multiple regions. The unmedicated group showed no significant differences in cortical thickness compared with HC. The results of three-group analysis of cortical thickness controlling for ICV, age and gender can be found in Supplementary Material, which were similar to the above findings. The details of the effects of diagnosis: BD/SZ on cortical thickness can be found in Supplementary Material. The results do not indicate diagnostic differences between BD and SZ with regard to the relationship between atypical antipsychotic treatment and cortical thickness alterations.

**TABLE 2:** Cortical regions with significant difference in cortical thickness among healthy controls, the medicated patients, and the unmedicated patients.

Brain region	Cluster size (mm^2^)	Talairach Coordinates (Peak Vertex)			*p-*values
**Left hemisphere**					
Inferior temporal gyrus	546.49	–53.4	–19.2	–30.3	<0.001
Insula	354.44	–33.7	14.9	13	<0.001
Superior frontal gyrus	339.42	–6.8	37.9	46.6	<0.001
Superior temporal gyrus	331.48	–47.6	4.8	–27.9	0.001
Isthmus cingulate gyrus	224.27	–12.3	–40.8	33.6	0.009
**Right hemisphere**					
Middle temporal gyrus	1090.79	54.4	3.4	–31.2	<0.001
Insula	736.88	32.2	9.4	9.2	<0.001
Lateral orbitofrontal cortex	480.16	33.3	31.3	–11.8	<0.001
Precentral gyrus	387.28	24.1	–13.8	63.8	<0.001
Isthmus cingulate gyrus	344.42	4.4	–42.2	29.9	<0.001
Superior parietal cortex	321.1	25.1	–60.5	33	<0.001
Superior frontal gyrus	268.73	12.7	19.8	37.4	0.003
Superior temporal gyrus	229.69	57.1	–12.5	–4.7	0.008

Multiple regression analyses in patients showed that diagnosis (BD/SZ) did not have significant effects on cortical thinning in all regions after FDR correction. Age had significant effects on cortical thinning in all regions (−0.008 to −0.003 mm/year) except the right middle temporal gyrus. Gender did not have significant effects on cortical thinning in all regions. Atypical antipsychotic use had significant effects on cortical thinning in all regions (*b* = −0.177 to −0.060 mm/year). Illness duration did not have significant effects on cortical thinning in all regions (Supplementary Table 4). We also used these possible confounding factors as covariates to observe the effect on the results. Potential confounding factors [diagnosis: BD/SZ, age, gender, duration of illness, antidepressants (yes/no) and anticonvulsants (yes/no)] had little impact on the results (Supplementary Figures 1–3). Additionally, higher olanzapine dose equivalents were significantly correlated with cortical thinning in the left superior temporal gyrus (*r* = −0.362, *p* = 0.011) after FDR correction. There was no significant correlation between duration of antipsychotic treatment and cortical thickness.

Regarding clinical symptoms, the HAMD scores of patients were significantly positively correlated with cortical thinning in the left isthmus of the cingulate cortex and the right middle temporal gyrus, right lateral orbitofrontal cortex, left superior temporal gyrus and left inferior temporal gyrus. The HAMA total score of patients was significantly positively correlated with cortical thinning in the left isthmus of the cingulate cortex, the right middle temporal gyrus and the left superior temporal gyrus. The YMRS total score in patients was significantly negatively correlated with cortical thinning in the left superior frontal gyrus. BPRS scores were significantly positively correlated with cortical thinning in the bilateral insula, the bilateral isthmus of the cingulate cortex, the left superior frontal gyrus, the left superior temporal gyrus, the right precentral gyrus, the left inferior temporal gyrus, the right middle temporal gyrus, the right lateral orbitofrontal cortex and the right superior parietal gyrus. Regarding cognitive function, WCST deficits in patients were significantly correlated with cortical thinning (correct responses and completed categories were positively correlated with cortical thickness; total errors, perseverative errors, and non-perseverative errors were negatively correlated with cortical thickness) in the right superior frontal gyrus, the left isthmus of the cingulate cortex, the right middle temporal gyrus, the right lateral orbitofrontal cortex and the left superior temporal gyrus. All the results above were set at *p* < 0.05 uncorrected, and the *p* and r values are listed in Table 3. After FDR correction, there was still a significant positive correlation between cortical thickness in the right middle temporal gyrus and the HAMD total (*r* = 0.283, *p* = 0.026), somatic anxiety (*r* = 0.295, *p* = 0.030), and psychic anxiety scores (*r* = 0.263, *p* = 0.041). The results of exploratory partial correlation analyses controlling for age, gender and illness duration can be found in Supplementary Table 5.

**TABLE 3:** Relationship between cortical thickness and clinical symptoms or cognitive function in all patients (medicated and unmedicated).

Regions variables	SPG.R	ICG.R	mSFG.R	ICG.L	PreCG.R	INS.R	INS.L	MTG.R	OFC.R	STG.L	ITG.L	mSFG.L
**Clinical symptoms**												
**HAMD total score**								0.283/ <0.001***	0.181/0.034*	0.186/0.030*	0.210/0.014*	
HAMD factor scores											0.209/0.014*	
Somatic anxiety								0.295/ <0.001***	0.181/0.034*	0.206/0.016*	0.232/0.006**	
Psychic anxiety								0.263/ 0.002**	0.204/0.017*		0.186/0.030*	
Core depressive				0.186/0.029*				0.201/0.019*	0.182/0.034*	0.172/0.045*		
Anorexia												
HAMA total score								0.225/0.011*		0.195/0.028*	0.185/0.037*	
**YMRS total score**												−0.200/0.028*
BPRS total score				0.211/0.011*								
**BPRS factor scores**												
Anxiety and depression								0.179/0.032*	0.192/0.021*			
Lack of energy	0.185/0.026*											
Thought disorder					0.173/0.038*							
Activity												
Hostility		0.178/0.033*		0.256/0.002**	0.183/0.028*	0.267/0.001**	0.226/0.006**		0.197/0.018*	0.182/0.029*	0.224/0.007**	0.297/0.018*
**Cognitive function**												
*WCST*												
Correct responses			0.205/0.043*	0.253/0.012*				0.268/0.008**	0.213/0.036*			
Categories completed			0.218/0.031*	0.227/0.025*						0.205/0.034*		
Total errors			−0.205/0.043*	−0.253/0.012*				−0.268/0.008**	−0.213/0.036*			
Perseverative errors								−0.218/0.032*				
Non-perseverative errors			−0.230/0.023*									

Exploratory partial correlation analyses controlling for age and sex were performed to determine the relationship between cortical thickness and clinical symptoms (HAMD total scores and its factors; HAMA total scores; YMRS total scores; and BPRS total scores and its factors) and cognitive function (WCST scores) separately. Data were presented as r-value/uncorrected p value; *** p < 0.001, ** p < 0.01, * p < 0.05. Bold indicates significance at p < 0.05, after FDR correction. R, right; L, left; SPG; superior parietal gyrus; ICG, isthmus cingulate gyrus; mSFG, medial superior frontal gyrus; PreCG, precentral gyrus; INS, insula; MTG, middle temporal gyrus; lOFC, lateral orbitofrontal Cortex; STG, superior temporal gyrus; ITG, inferior temporal gyrus.

### Age-Related Differences in Cortical Morphology

**FIGURE 3:**
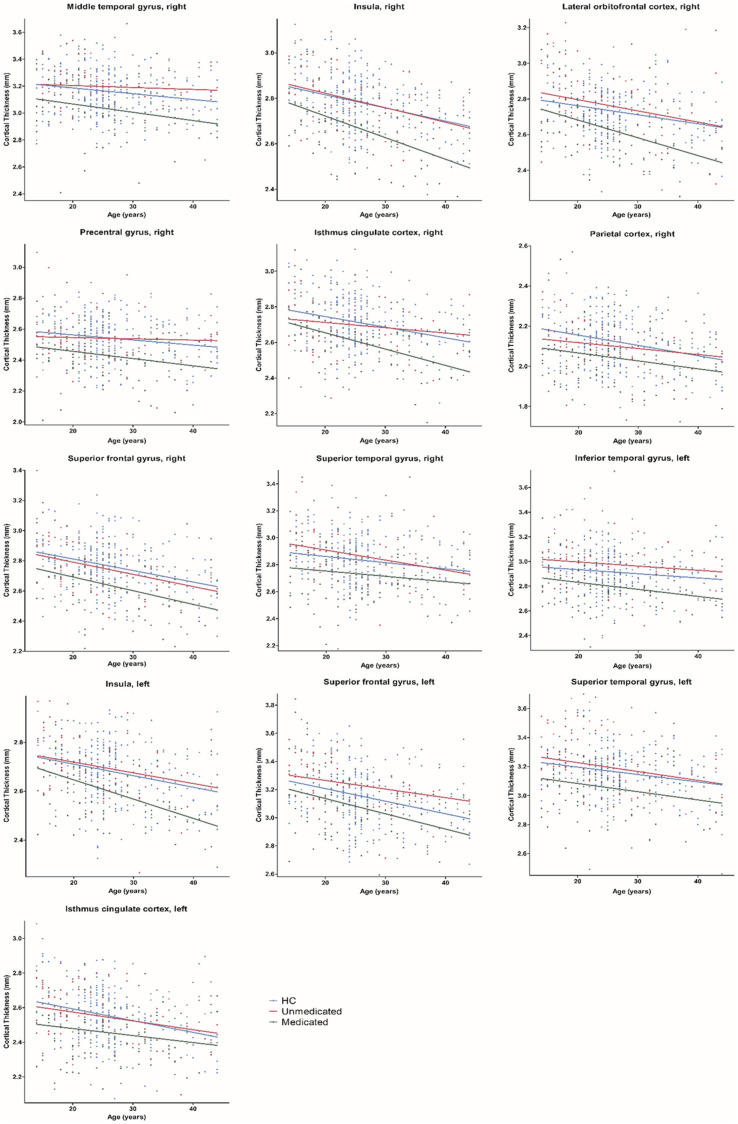
Age-related differences in cortical thickness among healthy controls, medicated patients, and unmedicated patients. HC, healthy controls.

Age-related effects on cortical thickness were observed in all 13 regions, showing group-level differences after FDR correction (Figure 3). *Post hoc* analyses showed that medicated patients had greater age-related cortical thinning in most (9 of 13) of these regions than did the unmedicated patients and HC (Table 4).

**TABLE 4:** Average rate of change in cortical thickness per year for healthy controls, the medicated patients, and the unmedicated patients.

Brain region	HC (mm/year)	Medicated (mm/year)	Unmedicated (mm/year)	Medicated vs. Unmedicated	Medicated vs. HC
**Left hemisphere**					
Inferior temporal gyrus	−0.003	−0.006	−0.003	↓	↓
Insula	−0.005	−0.008	−0.004	↓**	↓***
Superior frontal gyrus	−0.009	−0.011	−0.006	↓*	↓***
Superior temporal gyrus	−0.005	−0.006	−0.006	↑*	↓**
Isthmus cingulate gyrus	−0.007	−0.004	−0.005	↑	↑***
**Right hemisphere**					
Middle temporal gyrus	−0.004	−0.006	−0.001	↓**	↓***
Insula	−0.006	−0.010	−0.006	↓***	↓***
Lateral orbitofrontal cortex	−0.005	−0.010	−0.006	↓*	↓***
Precentral gyrus	−0.003	−0.005	−0.001	↓	↓***
Isthmus cingulate gyrus	−0.006	−0.009	−0.003	↓*	↓***
Superior parietal cortex	−0.005	−0.004	−0.003	↓	↑**
Superior frontal gyrus	−0.008	−0.009	−0.008	↓***	↓***
Superior temporal gyrus	−0.005	−0.004	−0.007	↑	↑

HC, healthy controls; the significance level was set at p < 0.05 with FDR correction for multiple comparison. ***p < 0.001, **p < 0.01, *p < 0.05. ↓, greater age-related thickness thinning in medicated patients compared to unmedicated patients or HC; ↑, smaller age-related thickness thinning in medicated patients compared to unmedicated patients or HC.

When the same effects were examined in relation to illness duration, no illness duration effects were found.

## Discussion

In this study, we found significant cortical thickness differences in the frontal, temporal, parietal, cingulate gyrus isthmus and insula areas among the three groups, with medicated patients showing significant cortical thinning. No differences in cortical surface area were found among the three groups. Furthermore, there were age-related differences in the effects of atypical antipsychotics in most (9 of 13) regions in the medicate group compared to the unmedicated and HC groups.

These findings were not related to illness duration. In an exploratory correlation analysis, cortical thinning in most regions was significantly positively correlated with lower HAMD, HAMA, and BPRS scores and WCST deficits (*p* < 0.05, uncorrected). After FDR correction, cortical thinning in the right middle temporal gyrus in patients was significantly positively correlated with lower HAMD total, somatic anxiety, and psychic anxiety scores. Finally, unmedicated patients had greater cortical thickness than HC only in the frontotemporal region.

In this study, cortical thinning in medicated patients relative to unmedicated patients provided preliminary evidence indicating negative effects of mid- to long-term atypical antipsychotic treatment on cortical thickness. Our results were consistent with studies showing the contribution of atypical antipsychotics to cortical thinning in the frontal, temporal, and parietal lobes, which has been repeatedly reported (Lesh et al., 2015; Zhang et al., 2018; Guo et al., 2019; Liu et al., 2020), and in the lateral orbitofrontal cortex (Gjerde et al., 2018; Akudjedu et al., 2020). Mid- to long-term treatment effects may be negative as a result of cumulative pharmacologic effects (Xiao et al., 2018). A study of BD published in 2020 found that patients currently being treated with atypical antipsychotics had significantly reduced total gray matter volumes compared to patients who were not taking atypical antipsychotics and to HC, which is similar to our results (Birner et al., 2020). The possible mechanism for cortical thinning may be that long-term atypical antipsychotic treatment could cause some loss of neurites, synaptic spines, or synapses in the cortical structures (Huang and Song, 2019). Animal studies have also shown an association between long-term exposure to atypical antipsychotics and reduced cortical volume (Dorph-Petersen et al., 2005). The atypical antipsychotic medication olanzapine was shown to exhibit neurotoxic effects by influencing autophagy (Vucicevic et al., 2014).

Our results showed greater age-related cortical thinning in medicated patients than in unmedicated patients and HC. The results were consistent with those of a few studies that did not differentiate between the effects of typical and atypical antipsychotics (van Haren et al., 2011; Alexander-Bloch et al., 2014) on cortical thinning and contradict the absence of an effect of antipsychotics on the age-related trajectory of cortical measures. We cannot deny that psychiatric disorders are progressive brain diseases that cause changes in cortical structures (van Haren et al., 2012). However, when illness duration was used as a covariate, all significant regions except the right parietal cortex survived. Moreover, we did not find evidence of an interaction between illness duration and group. Thus, we consider that the effect of atypical antipsychotics on thickness was greater than the effect of the disease itself (Fusar-Poli et al., 2013; Birner et al., 2020).

One possible reason for the occurrence of cortical thinning but not surface area reduction is that they have different neural mechanisms. In studies examining the role of genetic and environmental factors in thickness and surface area in a sample of 1,237 healthy adult twins, genetic factors contributed to approximately 45% of the variance in thickness (Kremen et al., 2010) but as much as 70% of the variance in surface area (Eyler et al., 2011). These results suggest that of these two structural measures, the brain plasticity caused by environmental influences (atypical antipsychotics, in our study) may be mainly reflected in changes in cortical thickness.

We observed a complex relationship between cortical structural changes and clinical features following mid- to long-term treatment. In this study, cortical thinning was associated with clinical improvement and, possibly, worsening cognitive function, suggesting atypical antipsychotics have both adaptive and maladaptive compensatory effects. Findings related to cognitive function were not significant after FDR correction, and further studies are needed to clarify the role of atypical antipsychotics in cortical thinning and subsequent changes in cognitive function. Worsening cognitive function may relate to dopamine sensitivity in psychosis and negative prognosis in patients receiving long-term, 7–20 years of antipsychotic treatment (Harrow and Jobe, 2018). Prior studies have generally indicated cortical volume loss during the course of illness in SZ; however, there are inconsistencies in findings such as the association of cortical structural loss with improved and worsening clinical severity (Gur et al., 1998; Sporn et al., 2003; Xiao et al., 2015; Walton et al., 2017; Moser et al., 2018; Guo et al., 2019). Several studies have found that atypical antipsychotics were associated with concurrent cortical structural loss and clinical improvement (Ahmed et al., 2015; Lesh et al., 2015; Guo et al., 2019). Further, atypical antipsychotics may have mechanistic role in cortical thinning as well as clinical improvement. Interestingly, greater cortical volume reduction in medicated adults and adolescents with SZ has been associated with greater clinical improvement at 3-year follow-up independent of medication types or baseline or follow-up clinical severity (Gur et al., 1998; Sporn et al., 2003). Some data found a negative correlation between cortical volume and regional homogeneity (ReHo) in the right inferior temporal gyrus in drug-naïve SZ but not in HC, suggesting that cortical volume reductions could be associated with increased ReHo (greater brain network integration) in SZ (Hong et al., 2019). In addition, cortical thinning has also been observed in high-risk people without disease onset (Bois et al., 2015). Altogether, these studies suggest that cortical thinning in medicated patients represent a potential compensatory mechanism associated with clinical improvement. Conceivably, cortical thinning could occur through known compensatory synaptic and cellular pruning of malfunctioning neurons. However, atypical antipsychotics are unlikely able to correct or arrest cortical defects and their progression that are already present.

Interestingly, we also observed more thickness in the left superior frontal gyrus and the inferior temporal gyrus in unmedicated patients than in medicated patients and HC. Greater thickness in these regions may be related to insufficient synaptic pruning during the neurodevelopmental process in SZ (Xiao et al., 2015). Moreover, we observed a trend of greater cortical thickness in the forebrain and less cortical thickness in the hindbrain in unmedicated patients than in HC. This is in line with the low-frequency fluctuation (ALFF) and regional homogeneity (ReHo) results of previous large-sample functional studies conducted by our group (Wei et al., 2018; Chang et al., 2019), suggesting that there are disease-related physiological imbalances (such as functional imbalances) that lead to cortical structural changes through some mechanisms. The negative findings of disease effects on surface area may be because the surface area is a weak intermediate phenotype for psychiatric disorders (Neilson et al., 2019). Our sample size is not adequate to identify disease-related differences.

There are several limitations to our study. First, the present study was conducted in a real-world context. Most patients were treated with more than one medication, and it was difficult to identify patients treated with a single medication. Therefore, it is unclear whether specific atypical antipsychotics cause cortical structure abnormalities. There may be interactions between different medications, a possibility that requires further investigation. Additionally, we note that the illness duration differed significantly between the medicated and unmedicated groups. However, this study demonstrated no effect of illness duration on the results. The multiple regression analysis and lack of interaction between illness duration and thickness argued against the possibility that our results were due to differences in illness duration between groups. Finally, given our cross-sectional design, the age-related results provided only a preliminary exploration of the cortical structural changes associated with atypical antipsychotics. However, it has been noted that most accelerated aging studies are cross-sectional designs. A longitudinal, placebo-controlled randomized controlled trial (RCT) design would be better, but patients in such studies would not be representative. Our real-world observations can provide complementary information and are more representative of the real-world situation. Second, our sample size was moderate and had a relatively wide age range (13–45 years). The broad age range and cross-sectional design may have limited the interpretation of our findings. Finally, the correlation findings are tentative because the uncorrected p and r values were generally modest, indicating modest effects.

Our major findings suggest that mid- to long-term atypical antipsychotic treatment is related to regional cortical thinning but not to a reduction in surface area and is associated with cognitive impairment. Furthermore, we provide the first evidence of age-related differences in the effects of mid- to long-term atypical antipsychotic treatment on cortical thickness in SZ and BD, suggesting that mid- to long-term atypical antipsychotic treatment may have negative effects.

## Data Availability Statement

Requests to access the datasets should be directed to FW, fei.wang@cmu.edu.cn.

## Ethics Statement

The studies involving human participants were reviewed and approved by ethics committee of the Institutional Review Board of China Medical University. Written informed consent to participate in this study was provided by the participants’ legal guardian/next of kin.

## Author Contributions

X-NZ, YT, and FW designed the study and wrote the protocol. SL, XZ, SW, XJ, CL, and SR acquired the data. YSW, MC, ZY, YGW, JD, and ZL analyzed the data. RF, FW, EE, and YC wrote the article. All authors contributed to and approved the final manuscript.

## Conflict of Interest

The authors declare that the research was conducted in the absence of any commercial or financial relationships that could be construed as a potential conflict of interest.

## Footnotes

1 https://github.com/zuoxinian/CCS

2 https://surfer.nmr.mgh.harvard.edu
