# Supplementary material for: Antipsychotic Effects on Cortical Morphology in Schizophrenia and Bipolar Disorders
Source: Front Neurosci. 2020 Dec 10;14:579139. doi: 10.3389/fnins.2020.579139 (PMC7758211; doi:10.3389/fnins.2020.579139)
Supplement: Supplementary file 1 [file Table_1.pdf]

Supplementary Material

1. Methods: Statistical Analyses

1.1 Confounding Factors

Analyses of covariance were performed using SPSS software. The possible confounding factors of age and gender were investigated as covariates for all groups, and diagnosis, duration of illness, antidepressants (yes/no) and anticonvulsants (yes/no) were investigated as covariates for the patient groups. The results were corrected using the FDR method, with $p < 0.05$.

1.2 Subgroup Analyses

Post hoc analyses of subgroups were performed using SPSS software. The results were corrected using the FDR method, with $p < 0.05$.

The effects of other medications (antidepressants and anticonvulsants) were assessed by comparing the cortical thickness of the antidepressant unmedicated and medicated subgroups or the anticonvulsant unmedicated and medicated subgroups separately.

Although it was not a primary aim of this investigation, the effects of diagnosis were assessed by comparing the cortical thickness of the BD and SZ subgroups of the unmedicated and medicated groups separately. Within the unmedicated group, 67 patients had SZ, and 21 had BD. Within the medicated group, 50 had SZ, and 34 had BD. Demographic and clinical details of the SZ and BD subgroups are presented in Supplementary Table 1.

1.3 Additional Whole-Brain Cortical Thickness Vertex-Based Analysis (All Participants Ages 18-45 years)

A three-group (HC, unmediated and medicated) analyses of cortical thickness in participants aged 18-45 were performed in FreeSurfer using ANCOVA, with group as an independent factor and age and gender as covariates. Statistical significance was determined by a vertex-level statistical threshold ($p < 0.001$) with Monte Carlo clusterwise simulation correction ($p < 0.01$) for multiple comparisons. Post hoc analyses were performed by permutation t-tests, and statistical significance was determined as $p < 0.05$ (FDR corrected).

1.4 Additional Whole-Brain Cortical Thickness Vertex-Based Analysis (controlling for ICV, Age and Gender)

A three-group (HC, unmediated and medicated) analyses of cortical thickness in participants were performed in FreeSurfer using ANCOVA, with group as an independent factor and ICV, age and gender as covariates. Statistical significance was determined by a vertex-level statistical threshold ($p < 0.001$) with Monte Carlo clusterwise simulation correction ($p < 0.01$) for multiple comparisons. Post hoc analyses were performed by permutation t-tests, and statistical significance was determined as $p < 0.05$ (FDR corrected).

1.5 Additional Whole-Brain Cortical Thickness Vertex-Based Analysis Between Diagnostic Subgroups

Two-sample t-tests analyses of cortical thickness between (A) medicated SZ and medicated BD patients and (B) unmedicated SZ and unmedicated BD patients were performed in FreeSurfer, with group as an independent factor and age and gender as covariates. Statistical significance was determined by a vertex-level statistical threshold ($p < 0.001$) with Monte Carlo clusterwise simulation correction ($p < 0.01$) for multiple comparisons.

2. Results

2.1 Confounding Factors Analyses

In general, the strength of relationships remained the same after controlling for age, gender, duration of illness, and antidepressant and anticonvulsant effects.

2.2 Subgroup Analyses

Significantly increased cortical thickness was found only in the left isthmus of the cingulate gyrus in the antidepressant medicated subgroup compared to the antidepressant unmedicated subgroup for regions showing significant group differences in cortical thickness ($p = 0.003$, FDR corrected). For the other 12 regions, there were no significant differences between antidepressant medicated and unmedicated subjects (Supplementary Figure 1).

Significantly decreased cortical thickness was found only in the left superior parietal gyrus in the anticonvulsant medicated subgroup compared to the anticonvulsant unmedicated subgroup for regions showing significant group differences in cortical thickness ($p = 0.0008$, FDR corrected). For the other 12 regions, there were no significant differences between anticonvulsant medicated and unmedicated subjects (Supplementary Figure 2).

No significant difference was observed between the SZ and BD subgroups of the unmedicated group for regions showing significant group differences in cortical thickness; no significant difference was observed between the SZ and BD subgroups of the medicated group for regions showing significant group differences in cortical thickness (Supplementary Figure 3).

2.3 Additional Whole-Brain Cortical Thickness vertex-based analysis (All Participants Ages 18-45 Years)

A three-group analysis of cortical thickness with a significance level set at $p < 0.01$ (Monte Carlo clusterwise simulation corrected, corresponding to $p < 0.001$, uncorrected) showed 13 regions with significant group differences. Post hoc analysis showed that compared to the unmedicated group and the HC, the medicated group had cortical thinning in the bilateral isthmus of the cingulate cortex, left inferior temporal gyrus, left insula, right middle temporal gyrus, right superior parietal gyrus, right lateral orbitofrontal cortex and right superior frontal gyrus. Compared to the HC group, the unmedicated group had increased cortical thickness in the left inferior temporal gyrus (Supplementary Figure 4).

2.4 Additional Whole-Brain Cortical Thickness vertex-based analysis (Controlling For ICV, Age and Gender)

A three-group analysis of cortical thickness included ICV, age and gender as covariates with a significance level set at $p < 0.01$ (Monte Carlo clusterwise simulation corrected, corresponding to $p < 0.001$, uncorrected) showed 8 regions with significant group differences. Compared to the unmedicated and HC groups, the medicated group had cortical thinning in the bilateral superior frontal gyrus, bilateral orbitofrontal cortex, left inferior temporal gyrus, left superior temporal gyrus, right insula and right middle temporal gyrus. Compared to the HC, the unmedicated group had

increased cortical thickness in the left inferior temporal gyrus and the bilateral superior frontal gyrus (Supplementary Figure 5).

2.5 Additional Whole-Brain Cortical Thickness Vertex-based Analysis Between Diagnostic Subgroups

There were no significant differences in cortical thickness between medicated SZ patients and medicated BD patients. The unmedicated SZ patients only showed increased cortical thickness in the left superior frontal gyrus compared to the unmedicated BD patients (Supplementary Figure 6).

Supplementary Table 1. Demographic, clinical characteristics and cognitive function of the SZ and BD medicated patients, and the SZ and BD unmedicated patients

Variable	Medicated patients (n=88)		Unmedicated patients (n=84)	
	SZ (n=67)	BD (n=21)	SZ (n=50)	BD (n=34)
**Demographic characteristic**				
Age (years)	24.91 (8.48)	27.24 (6.70)	25.54 (9.46)	24.47 (5.95)
Male	26 (39%)	12 (57%)	22 (44%)	12 (35%)
Right handedness	56 (84%)	21 (100%)	42 (84%)	30 (88%)
**Clinical characteristic**				
Illness duration (months)	49.88 (51.39)	88.94 (74.44)	10.38 (21.77)	25.35 (33.95)
First episode, yes	33 (49%)	6 (29%)	43 (86%)	22 (65%)
HAMD Total	(n=57)	(n=19)	(n=34)	(n=31)
	4.84 (5.00)	7.37 (8.35)	11.82 (8.15)	15.13 (10.53)
HAMA Total	(n=50)	(n=19)	(n=32)	(n=28)
	3.56 (3.49)	4.74 (5.18)	10.78 (8.47)	11.11 (9.72)
YMRS Total	(n=44)	(n=19)	(n=32)	(n=28)
	1.09 (3.82)	6.89 (10.00)	1.94 (4.85)	7.00 (11.38)
BPRS Total	(n=64)	(n=14)	(n=47)	(n=21)
	29.02 (10.48)	25.21 (9.86)	38.91 (11.67)	24.67 (7.61)
**Cognitive function**				
WCST	(n=39)	(n=14)	(n=26)	(n=21)
Corrected responses	17.28 (10.47)	27.79 (10.43)	21.46 (12.93)	26.86 (11.88)
Categories completed	1.72 (2.15)	3.71 (1.82)	2.19 (2.02)	3.24 (2.07)
Total errors	30.72 (10.47)	20.21 (10.43)	26.54 (12.93)	21.14 (11.88
Perseverative errors	12.41 (8.98)	6.43 (4.01)	11.31 (12.83)	7.67 (9.15)
Non-perseverative errors	18.31 (7.57)	13.79 (7.31)	15.23 (8.64)	13.57 (8.08)
**Medication**				
Antipsychotic use c				
Duration (months)	21.18 (25.82)	17.72 (25.31)	-	-
Dose OPZ (mg)	6.60 (5.72)	6.41 (5.35)	-	-
Antipsychotic type				
Amisulpride	3 (4%)	0	-	-

Aripiprazole	21 (31%)	3 (14%)	-	-
Clozapine	12 (18%)	0	-	-
Olanzapine	12 (18%)	5 (24%)	-	-
Paliperidone	2 (3%)	0	-	-
Quetiapine	8 (12%)	9 (43%)	-	-
Risperidone	29 (43%)	4 (19%)	-	-
Ziprasidone	3 (4%)	0	-	-
Antidepressants (%)	9 (13%)	6 (33%)	-	-
Anticonvulsants (%)	3 (4%)	14 (66%)	-	-

Data were presented as either n (%) or mean (SD). HC, Healthy Controls; HAMD, Hamilton Depression Scale; HAMA, Hamilton anxiety Scale; YMRS, Young Mania Rating Scale; BPRS, Brief Psychiatric Rating Scale; WCST, Wisconsin Card Sorting Test. OPZ, Olanzapine equivalent value.

Supplementary Table 2. Cortical regions with significant difference in cortical thickness between the medicated patients and healthy controls

Brain region	Cluster size (mm^2)	Talairach Coordinates (Peak Vertex)			*p* values
**Left hemisphere**					
Inferior temporal gyrus	829.68	-53.8	-21.1	-29.7	< 0.001
Insula	396.07	-34.1	14.6	12.8	< 0.001
Isthmus cingulate gyrus	356.24	-12.9	-41.6	33.4	< 0.001
**Right hemisphere**					
Insula	1789.54	32.3	8.9	9.9	< 0.001
Superior temporal gyrus	946.22	49.6	1	-26.2	< 0.001
Precentral gyrus	557.57	20.4	-8.5	56.9	< 0.001
Isthmus cingulate gyrus	444.49	4.4	-42.9	29.6	< 0.001
Superior parietal cortex	421.19	24.7	-61.1	32.5	< 0.001
Superior frontal gyrus	339.7	12.7	19.8	37.4	< 0.001
Superior temporal gyrus	268.2	51.2	-16.6	-6.9	0.004
Fusiform gyrus	243.59	34.2	-70.3	-13	0.008

Supplementary Table 3. Cortical regions with significant difference in cortical thickness between the medicated patients and unmedicated patients

Brain region	Cluster size (mm^2)	Talairach Coordinates (Peak Vertex)			*p* values
**Left hemisphere**					
Inferior temporal gyrus	545.16	-53	-18.6	-30.1	< 0.001
Superior frontal gyrus	524.42	-6.9	37.1	46.6	< 0.001
Insula	299.71	-35	-3.4	15.7	0.003
**Right hemisphere**					
Middle temporal gyrus	914.58	43.2	11.3	-35.3	< 0.001
Insula	587.28	31.7	11.1	8.2	< 0.001
Lateral orbitofrontal cortex	414.31	34	31.3	-12.5	< 0.001
Superior frontal gyrus	303.97	8.8	54	25.6	< 0.001
Superior frontal gyrus	300.59	6.8	38.8	47.1	< 0.001

Supplementary Table 4. Multiple regression analyses: the influence of diagnosis: BD/SZ, age, gender, atypical antipsychotic use (yes/no) and illness duration on cortical thickness in patients

Brain region	diagnosis: BD/SZ (SZ=0, BD=1)		Age (years)		Gender (male=0, female=1)		Atypical antipsychotic (0=no, 1=yes)		Illness duration (years)	
	b	t (*p*)	b	t (*p*)	b	t (*p*)	b	t (*p*)	b	t (*p*)
**Left hemisphere**										
Inferior temporal gyrus	0.006	0.167 (0.867)	**-0.004**	**-2.061 (0.041)**	-0.082	-2.679 (0.008)	**-0.177**	**-5.112 (< 0.001)**	0.000	-0.095 (0.925)
Insula	-0.007	-0.384 (0.701)	**-0.006**	**-4.773 (< 0.001)**	-0.026	-1.465 (0.145)	**-0.082**	**-4.135 (< 0.001)**	-0.003	-1.203 (0.231)
Superior frontal gyrus	-0.078	-2.248 (0.026)	**-0.007**	**-3.638 (< 0.001)**	0.007	0.238 (0.812)	**-0.147**	**-4.146 (< 0.001)**	-0.005	-1.208 (0.229)
Superior temporal gyrus	0.023	0.692 (0.490)	**-0.006**	**-2.853 (0.005)**	-0.038	-1.260 (0.209)	**-0.137**	**-3.972 (< 0.001)**	0.000	-0.063 (0.950)
Isthmus cingulate gyrus	-0.033	-1.170 (0.244)	**-0.005**	**-2.903 (0.004)**	-0.010	-0.396 (0.693)	**-0.102**	**-3.553 (< 0.001)**	0.002	0.569 (0.570)
**Right hemisphere**										
Middle temporal gyrus	0.025	0.798 (0.426)	-0.003	-1.765 (0.079)	-0.018	-0.631 (0.529)	**-0.148**	**-4.633 (< 0.001)**	-0.003	-0.740 (0.460)
Insula	-0.015	-0.669 (0.504)	**-0.007**	**-5.592 (< 0.001)**	-0.012	-0.594 (0.533)	**-0.109**	**-4.784 (< 0.001)**	-0.003	-1.062 (0.290)
Lateral orbitofrontal cortex	-0.024	-0.803 (0.423)	**-0.008**	**-4.530 (<0.001)**	-0.019	-0.702 (0.484)	**-0.137**	**-4.478 (< 0.001)**	0.000	-0.061 (0.952)
Precentral gyrus	-0.055	-2.058 (0.041)	**-0.003**	**-2.097 (0.037)**	0.067	2.779 (0.006)	**-0.123**	**-4.485 (< 0.001)**	0.002	0.517 (0.606)
Isthmus cingulate gyrus	-0.009	-0.357 (0.721)	**-0.005**	**-3.327 (0.001)**	-0.001	-0.051 (0.959)	**-0.072**	**-2.738 (0.007)**	-0.006	-1.759 (0.080)
Superior parietal cortex	-0.047	-1.987 (0.049)	**-0.003**	**-2.274 (0.024)**	0.005	0.232 (0.817)	**-0.060**	**-2.469 (0.015)**	-0.001	-0.453 (0.651)
Superior frontal gyrus	-0.036	-1.247 (0.214)	**-0.008**	**-4.678 (< 0.001)**	0.030	1.151 (0.252)	**-0.097**	**-3.251 (0.001)**	-0.003	-0.925 (0.356)
Superior temporal gyrus	-0.023	-0.661 (0.510)	**-0.006**	**-2.768 (0.006)**	-0.048	-1.503 (0.135)	**-0.150**	**-4.112 (< 0.001)**	0.002	0.488 (0.627)

Note: Bold indicates significance at $p < 0.05$, FDR corrected.

Supplementary Table 5. **Relationship between cortical thickness and clinical symptoms or cognitive function in all patients (age, gender and duration of illness as covariates)**

**Variables / Regions**	**SPG.R**	**mSFG.R**	**ICG.L**	**PreCG.R**	**INS.R**	**INS.L**	**MTG.R**	**OFC.R**	**STG.L**	**ITG.L**	**mSFG.L**
**Clinical Symptoms**											
HAMD Total Score							0.236/0.006**				
HAMD Factor Scores											
Somatic anxiety							0.251/0.003**				
Psychic anxiety							0.222/0.009**	0.182/0.034*		0.179/0.037*	
Core depressive			0.178/0.038*								
Anorexia											
HAMA Total Score							0.197/0.027*				
YMRS Total Score											-0.196/0.032*
BPRS Total Score			0.194/0.020*								
BPRS Factor Scores											
Anxiety and depression								0.180/0.032*			
Lack of energy	0.174/0.038*										
Thought disorder				0.166/0.047*							
Activity											
Hostility			0.240/0.004*		0.231/0.005*	0.190/0.023*		0.182/0.029*		0.194/0.020*	
**Cognitive Function**											
WCST											
Correct Responses		0.216/0.034*	0.252/0.013*		0.202/0.048*		0.276/0.006**	0.217/0.033*			
Categories Completed		0.232/0.022*	0.225/0.026*					0.200/0.049*	0.219/0.031*		
Total Errors		-0.216/0.034*	-0.252/0.013*		-0.202/0.048*		-0.276/0.006**	-0.217/0.033*			
Perseverative Errors							-0.218/0.032*				

Non-perseverative Errors	-0.230/0.023*

Exploratory partial correlation analyses controlling for age and sex were performed to determine the relationship between cortical thickness and clinical symptoms (HAMD total scores and its factors; HAMA total scores; YMRS total scores; and BPRS total scores and its factors) and cognitive function (WCST scores) separately.

Note: Data were presented as r value/uncorrected *p* value; ***, $p < 0.001$. **, $p < 0.01$. *, $p < 0.05$. A significant result was not obtained at $p < 0.05$ after FDR correction. R, right; L, left; SPG; superior parietal gyrus; ICG, isthmus cingulate gyrus; mSFG, superior frontal gyrus; PreCG, precentral gyrus; INS, insula; MTG, middle temporal gyrus; lOFC, lateral orbitofrontal cortex; STG, superior temporal gyrus.

Supplementary Figure 1. Comparison of cortical thickness between antidepressant unmedicated and medicated subgroups

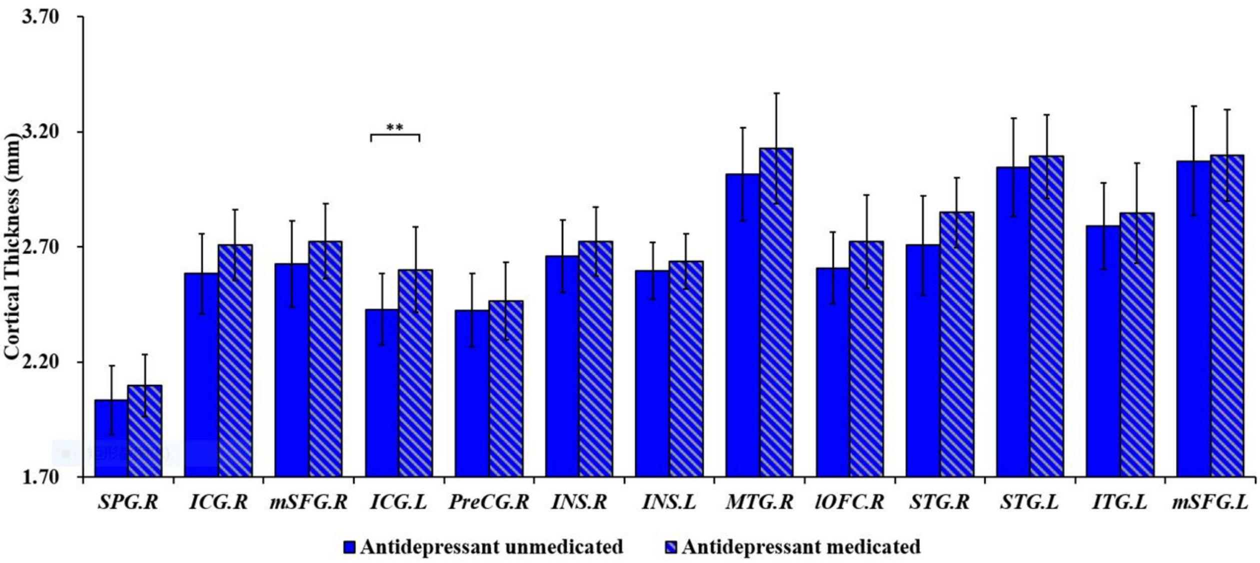

**, $p < 0.01$ after FDR correction.

R, right; L, left; SPG; superior parietal gyrus; ICG, isthmus cingulate gyrus; mSFG, superior frontal gyrus; PreCG, precentral gyrus; INS, insula; MTG, middle temporal gyrus; lOFC, lateral orbitofrontal cortex; STG, superior temporal gyrus.

Supplementary Figure 2. Comparison of cortical thickness between anticonvulsant unmedicated and medicated subgroups

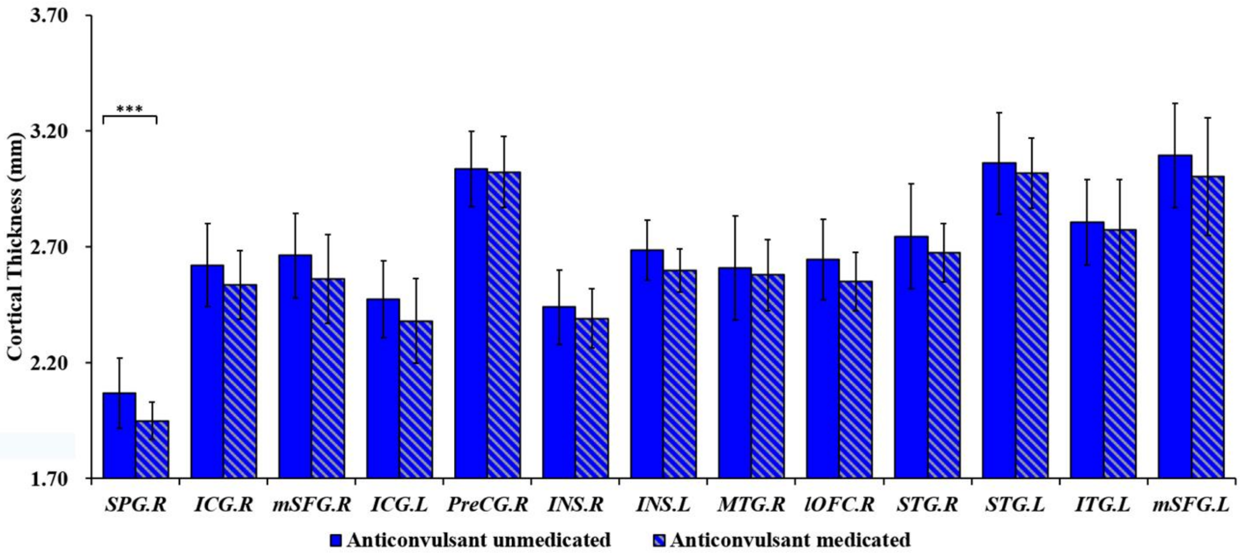

***, $p < 0.001$ after FDR correction.

R, right; L, left; SPG; superior parietal gyrus; ICG, isthmus cingulate gyrus; mSFG, superior frontal gyrus; PreCG, precentral gyrus; INS, insula; MTG, middle temporal gyrus; lOFC, lateral orbitofrontal cortex; STG, superior temporal gyrus.

Supplementary Figure 3. Comparison of cortical thickness between diagnostic subgroups (SZ unmedicated vs. BD unmedicated; SZ medicated vs. BD medicated)

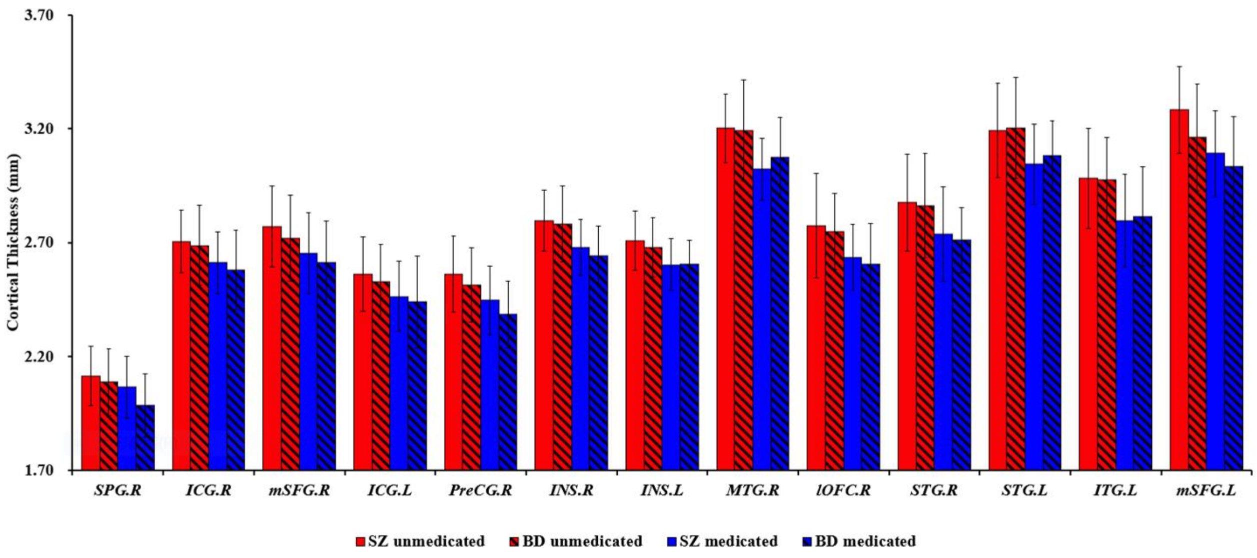

SZ, schizophrenia; BD, bipolar disorder; R, right; L, left; SPG; superior parietal gyrus; ICG, isthmus cingulate gyrus; mSFG, superior frontal gyrus; PreCG, precentral gyrus; INS, insula; MTG, middle temporal gyrus; lOFC, lateral orbitofrontal cortex; STG, superior temporal gyrus.

Supplementary Figure 4. Significant difference of cortical thickness among HC, the unmedicated patients, and the medicated patients (all participants ages 18-45)

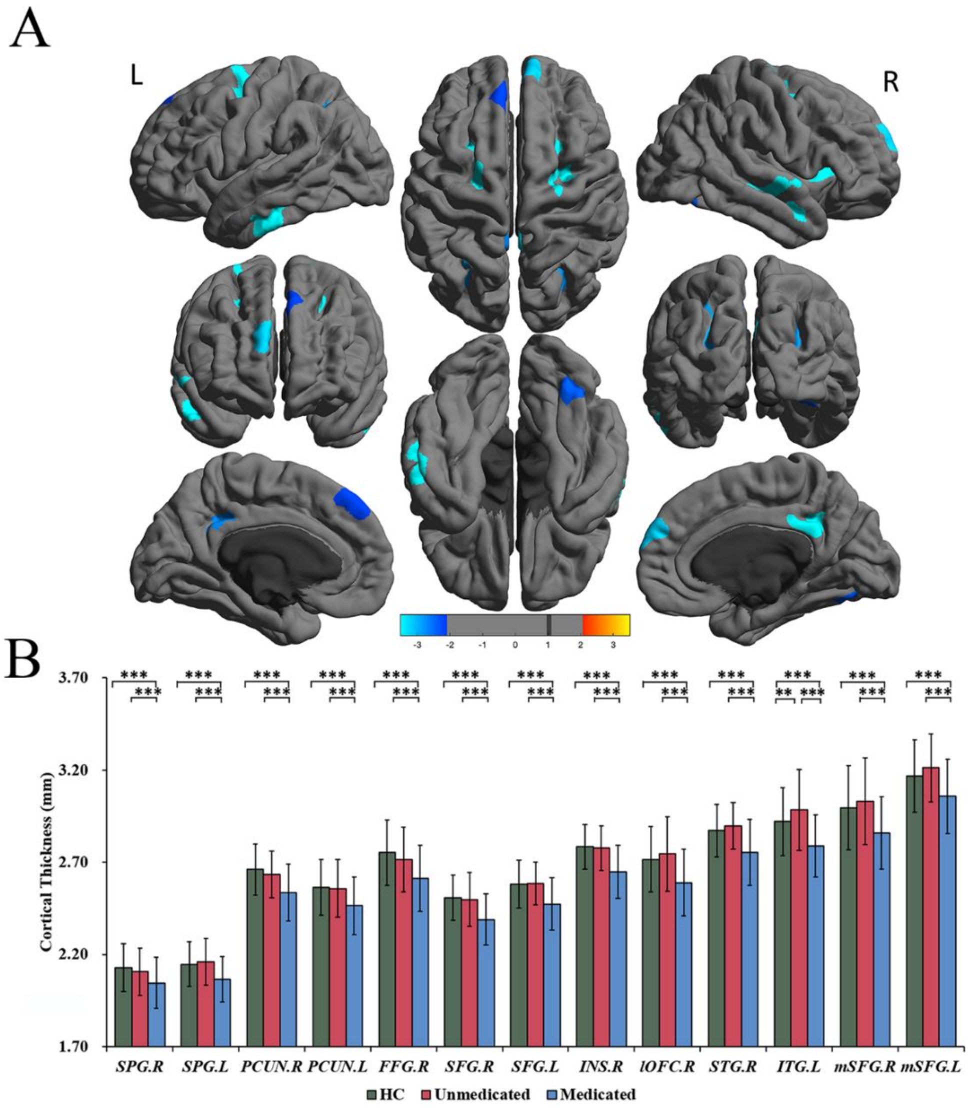

(A) Regions with significant thickness differences among the three groups. The significance level was set at $p < 0.001$ at the vertex level with Monte Carlo clusterwise simulation correction for multiple comparisons ($p < 0.01$, corrected). The colour bar represents the t value.

(B) Post hoc pairwise comparisons showing thickness differences between each pairing (HC vs. medicated, HC vs. unmedicated, medicated vs. unmedicated). The significance level was set at $p < 0.05$, with FDR correction for multiple comparisons. ***, $p < 0.001$. **, $p < 0.01$. *, $p < 0.05$.

HC, healthy controls; R, right; L, left; SPG; superior parietal gyrus; PCUN, precuneus; FFG, fusiform gyrus; mSFG, medial superior frontal gyrus; SFG, superior frontal gyrus; ISN, insula; lOFC, lateral orbitofrontal Cortex; STG, superior temporal gyrus; ITG, inferior temporal gyrus.

Supplementary Figure 5. Significant difference of cortical thickness among HC, the unmedicated patients, and the medicated patients (controlling for intracranial volume, age and gender)

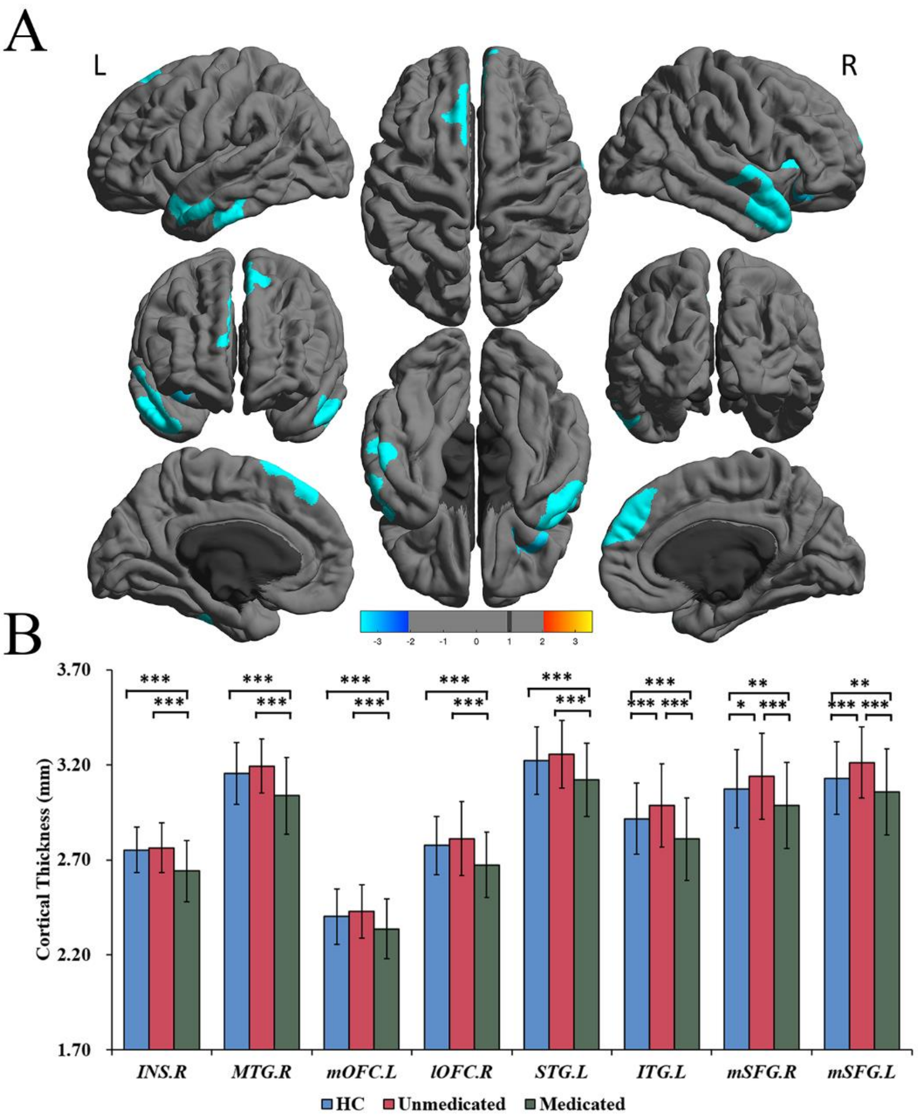

(A) Regions with significant thickness differences among three groups. The significance level was set to vertex $p < 0.001$ with Monte Carlo cluster-wise simulation correction for cluster $p < 0.01$. L, left. R, right. The color bar represents t value.

(B) Post-hoc pair-wise comparisons showing thickness differences between each pair group (HC vs. Medicated, HC vs. Unmedicated, Medicated vs. Unmedicated). The significance level was set at $p < 0.05$ with FDR correction for multiple comparison. ***, $p < 0.001$. **, $p < 0.01$. *, $p < 0.05$.

HC, healthy controls; R, right; L, left; ISN, insula; mOFC, medial orbitofrontal cortex; lOFC, lateral orbitofrontal Cortex; mSFG, medial superior frontal gyrus; STG, superior temporal gyrus; MTG, middle temporal gyrus; ITG, inferior temporal gyrus.

Supplementary Figure 6. Significant difference of cortical thickness between the unmedicated BD patients and the unmedicated SZ patients

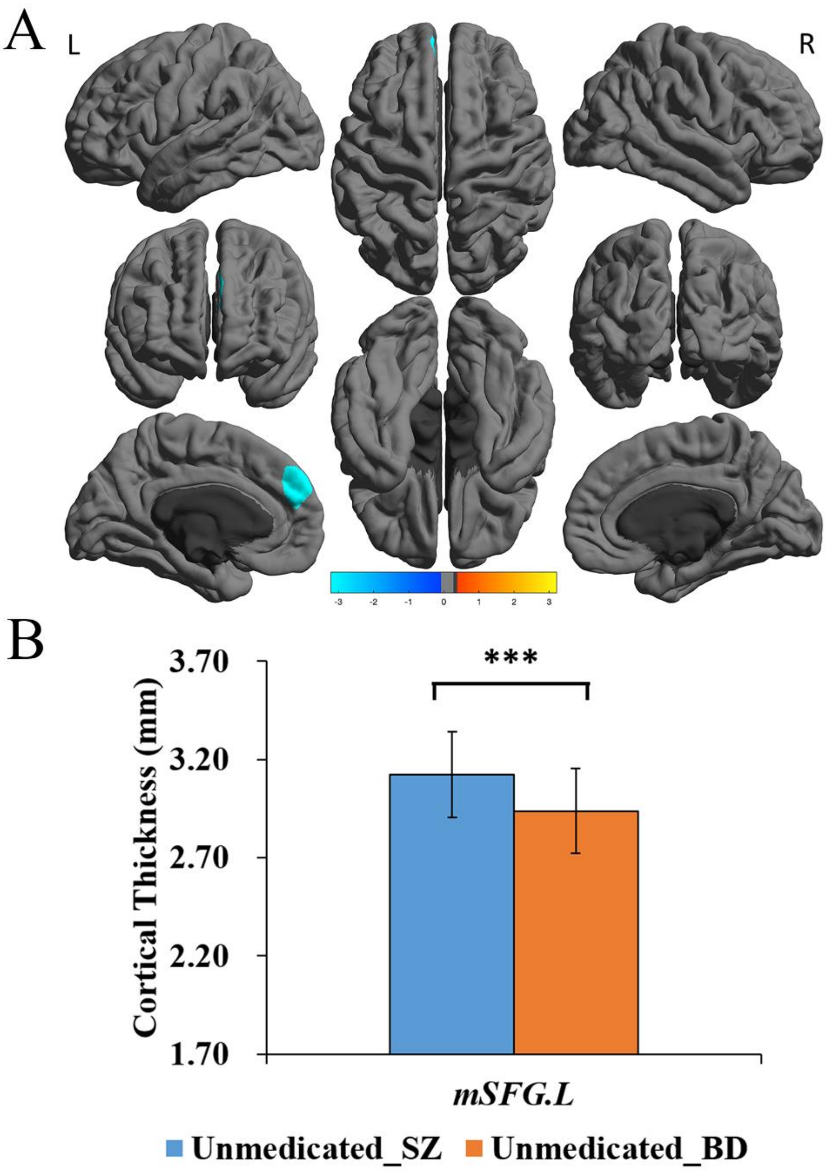

(A) Regions with significant thickness differences between groups. The significance level was set to vertex $p < 0.001$ with Monte Carlo cluster-wise simulation correction for cluster $p < 0.01$. L, left. R, right. The color bar represents t value.

(B) Post-hoc pair-wise comparisons showing thickness differences between groups. The significance level was set at $p < 0.05$ with FDR correction for multiple comparison. ***, $p < 0.001$.

SZ, schizophrenia; BD, bipolar disorder; L, left; mSFG, medial superior frontal gyrus.
